# Postoperative *Paenibacillus thiaminolyticus* Wound Infection, Switzerland

**DOI:** 10.3201/eid2707.203348

**Published:** 2021-07

**Authors:** Riccardo Di Micco, Matthias Schneider, Reto Nüesch

**Affiliations:** Spital Schwyz, Schwyz, Switzerland (R. Di Micco, M. Schneider, R. Nüesch);; University of Basel, Basel, Switzerland (R. Nüesch)

**Keywords:** *Paenibacillus thiaminolyticus*, MALDI-TOF, matrix-assisted laser desorption/ionization time-of-flight mass spectrometry, wound infection, bacteria, antimicrobial resistance, Switzerland

## Abstract

*Paenibacillus thiaminolyticus* is a nonvirulent organism found in human and ruminant microbiota. However, *P. thiaminolyticus* can act as an opportunistic pathogen in humans. We describe a case of abdominal wall hematoma secondarily infected by *P. thiaminolyticus*. Our findings emphasize the risk for unusual *Paenibacillus* infections in otherwise healthy persons.

The genus *Paenibacillus* comprises a growing number of species of rod-shaped, motile bacteria with peritrichous flagella (*1*). *Paenibacillus* species share 89.6% similarity of 16S rDNA gene sequences and grow as nonpigmented colonies on tryptic soy agar (*1*). Best known as a nearly ubiquitous environmental bacteria, many *Paenibacillus* species are potential opportunistic pathogens in humans (*2*). We report a case of isolated surgical site infection caused by *P. thiaminolyticus* in an otherwise healthy patient.

A 33-year-old woman came to the emergency department with a fever and reported having a painful and fluctuating abdominal wall mass for 3 days. She had undergone lipoabdominoplasty in a different hospital 7 days earlier. Laboratory tests showed anemia (hemoglobin 88 g/L, hematocrit 0.24 L/L) and isolated C-reactive protein elevation (117 mg/L). Computed tomography of the abdomen demonstrated a fluid collection in the abdominal wall measuring 22 × 9.5 × 5 cm. The patient was admitted for observation. Blood cultures performed at 38.5°C showed no bacterial growth. 

Empirical intravenous antimicrobial drug therapy for suspected infected hematoma was initiated with amoxicillin/clavulanate (2.2 g 3×/d), according to local hospital guidelines. Under antimicrobial drug treatment, the patient’s fever resolved, but her abdominal pain persisted. 

On day 3, we aspirated a sample of the fluid collection in the abdominal wall for microbiological examination. The aspirate was cultured on blood agar incubated at 35°C with 5% CO_2_ for 48 h; on MacConkey agar incubated at 35°C, aerobic, for 24 h; and on selective anaerobic agar at 35°C, anaerobic, for 5 days. All 3 yielded a pure culture of gram variable rod-shaped bacteria. We used Biotyper matrix-assisted laser desorption/ionization time-of-flight (MALDI-TOF) mass spectrometry (Bruker Corporation, https://www.bruker.com) and the Bruker mass spectra database, which returned *P. thiaminolyticus* with a best-match score of 2.07 (a score >2 means identification at the species level) (*3*,*4*). 

On day 7, the patient had bleeding at the surgical site, and we performed a surgical evacuation with drainage of the fluid collection. We took an intraoperative microbiological swab specimen and ran another MALDI-TOF mass spectrometry analysis, which confirmed the pathogen as *P. thiaminolyticus* with a best match score of 2.17. 

After evacuation of the hematoma, the patient rapidly recovered. Because no specific clinical breakpoints have been established for *Paenibacillus* spp., we used nonspecies related clinical breakpoints from the European Committee on Antimicrobial Susceptibility Testing pharmacokinetics and pharmacodynamics (Table). Intravenous antimicrobial drug therapy was continued for a total of 10 days. On day 14, the patient was discharged with oral amoxicillin/clavulanate (1 g 3×/d) for another 2 weeks. We decided to perform a clinical and laboratory follow up at 2, 4, and 8 weeks after discharge. After 2 months, the surgical wound had healed, and the patient was well and without sequelae.

**Table:** Comparison of MIC and SS of antimicrobial drugs in blood 4 hours after intravenous administration to treat *Paenibacillus thiaminolyticus* in a patient, Switzerland*

Antimicrobial drug†	MIC	SSC
Amoxicillin/clavulanate	0.064 mg/L	1.2 mg/L
Tetracycline	12 mg/L	2.8 mg/L
Trimethoprim/sulfamethoxazole‡	<0.094 mg/L	1.5–3 mg/L
Clindamycin	0.38 mg/L	23 mg/L
Ciprofloxacin	0.19 mg/L	4.56 mg/L

*No specific clinical breakpoints have been established for *Paenibacillus* spp. We used European Committee on Antimicrobial Susceptibility Testing pharmacokinetics and pharmacodynamics to determine antimicrobial susceptibility. SSC, steady state concentration.

†Antimicrobial drugs were administered at the recommended doses reported in the manufacturer the data sheets (compendium.ch, https://compendium.ch). ‡Trimethoprim/sulfamethoxazole is dose dependent.

Of the 49 species of *Paenibacillus* known to cause symptomatic infection in humans, the most commonly reported are *P. alvei*, *P. phoenicis*, *P. macerans*, *P. lautus*, *P. timonensis*, *P. provencensis*, and *P. thiaminloyticus* (*2*). Clinical manifestation in patients is heterogeneous, ranging from paucisymptomatic to severe sepsis. The bacteria usually are found in blood with manifest bacteremia (*2*). In this case, *P. thiaminolyticus* was found in the aspirates of the infected abdominal wall hematoma but not in blood cultures or other body compartments.

Because *Paenibacillus* spp. are possible laboratory contaminants (*5*), the organisms should be detected in multiple sets to rule out contamination. The absence of clear, discriminating phenotypical features calls for molecular biology methods to identify the bacterium, such as MALDI-TOF mass spectrometry or, when in doubt, 16S rRNA gene sequencing (*4*).

*P. thiaminolyticus* is reported as potentially resistant to ampicillin alone (*2*), vancomycin (*2*), and clindamycin (*6*). In this case, the bacterium showed tetracycline resistance. Consequently, antimicrobial susceptibility testing is necessary. According to the antibiograms reported in the literature, empiric therapy with trimethoprim/sulfamethoxazole or amoxicillin/clavulanate is recommended. Although this patient’s condition improved with intravenous antimicrobial drug therapy, clinical resolution occurred only after surgical evacuation of the abdominal wall fluid collection. Because of reports of persistent infections (*7*), patients should be monitored after treatment.

*P. thiaminolyticus* was identified in human feces in 1951 (*8*). Anecdotally, its thiaminase activity can reduce available thiamin necessary for energy metabolism in the central nervous system, causing poliencephalomalacia in ruminants (*9*). So far, no human disease syndrome has been related explicitly to *P. thiaminolyticus*. In 2008, *P. thiaminolyticus* was reported as the causative agent of bacteremia of unknown origin in a dialysis patient with multiple underlying conditions and a long-term catheter (*6*). Since then, 3 other isolates were reported in blood (*2*), vitreous humor (*2*), and cerebrospinal fluid (*10*).

In summary, this case is a reminder of the existence of a rare potential pathogen in our microbiota, although the causality might be discussed because *Paenibacillus* spp. remain mostly environmental bacteria. Therefore, identification relies on MALDI-TOF mass spectrometry or 16S rRNA gene sequencing. Surgical debridement of the infection focus also is recommended. The microorganism shows a variable antimicrobial susceptibility profile, and trimethoprim/sulfamethoxazole and amoxicillin/clavulanate are possible first choice empiric therapies after successful identification.
